# Neglected neurogenic clubfoot treated with Achilles tendon lengthening using Z-plasty, total talectomy, and tibiocalcaneal arthrodesis

**DOI:** 10.1016/j.ijscr.2021.106051

**Published:** 2021-06-02

**Authors:** Ihsan Oesman, Chintya Mutiara Sari

**Affiliations:** a Foot and Ankle Consultant, Department of Orthopaedic and Traumatology, Faculty of Medicine, Universitas Indonesia, Dr. Cipto Mangunkusumo National Central General Hospital, Jakarta, Indonesia; b Department of Orthopaedic and Traumatology, Faculty of Medicine, Universitas Indonesia, Dr. Cipto Mangunkusumo National Central General Hospital, Jakarta, Indonesia

**Keywords:** Neurogenic clubfoot, Achilles tendon lengthening, Total talectomy, Tibiocalcaneal arthrodesis

## Abstract

**Introduction:**

The most common foot and ankle deformity from injury to the nervous system is equinocavovarus. This deformity comprises of equinus, cavus, varus, and adduction of the forefoot which leads to pain and poor stability in stance phase of gait. Treatment for this condition is difficult regarding literature limitation of the neurogenic clubfoot management. We reported a 18-year-old female with neglected right neurogenic clubfoot treated with 2 stage deformity correction.

**Case report:**

A 18-year-old female presented with crooked right foot since birth. It caused pain, especially during walking and standing for a long time and resulted in occasional skin infection on the bottom of the foot. However, currently she could walk in limping gait without walking aid. The patient was born aterm 39 weeks through caesarean delivery due to severe preeclampsia. There was delayed development of walking at 2 years and 9 months. Previously, she had history of spina bifida and undergone surgery in 2001. Afterward, she underwent VP shunt surgery. Physical examination demonstrated cavus varus, tenderness of the right foot, and limited ankle motion. The patient was diagnosed with neglected right neurogenic clubfoot and underwent two stage deformity correction consisting of Achilles tendon lengthening using Z-plasty, total talectomy, and tibiocalcaneal arthrodesis followed by posteromedial release, tendon lengthening (Tibialis posterior, FDL, FDB) and plantar fascia release.

**Conclusions:**

Two stage deformity correction can be successful in patients with neglected neurogenic clubfoot. Further studies are required to investigate the safety and efficacy of such procedure in neurogenic clubfoot.

## Highlights

• Neglected right neurogenic clubfoot with history of preeclampsia during pregnancy
• History of spina bifida with developmental delay in gross motor
• Treated with deformity correction consisting of Achilles tendon lengthening using Z-plasty, total talectomy, and tibiocalcaneal arthrodesis followed by posteriomedial release, tendon lengthening (Tibialis posterior, FDL, FDB) and plantar fascia release

## 1 Introduction

Clubfoot occurs in approximately 1 of every 1000 live births, with bilateral deformities occurring in approximately 50% of these children [1]. Although congenital talipes equinovarus (CTEV) has many theories of etiology, no clearly defined etiological process has been agreed upon [2]. Multiple theories have been proposed regarding the cause of clubfoot. The congenital deformity can be further divided into idiopathic and nonidiopathic types, and the acquired deformity classified into neurogenic and vascular causes. The most common foot and ankle deformity from injury to the nervous system is equinocavovarus. This deformity is manifested as a combination of equinus, cavus, varus, and adduction of the forefoot. This leads to pain and poor stability in stance phase of gait. Treatment for this condition is difficult due to the paucity of literature regarding the management of the neurogenic equinovarus [3].

With more than two-thirds of all children lacking access to specialized orthopaedic care, neglected clubfoot is a common problem in resource poor environments [4]. While the patho-anatomy remains unchanged, the treatment becomes more complex with advancing age due to the severity of soft tissue contractures and the limited remodeling potential [4]. Neglected clubfoot can't be solved with only conservative treatment. Surgical treatment of neglected talectomy is necessary to provide the best in both clinical and functional outcome of the patient. Surgical treatment can be done with soft tissue procedure and bony procedure. Soft tissue procedure usually is performed using achilles tendon lengthening, however it may cause of equinovarus deformity relaps. Bony procedure is needed with the careful consideration [5]. The most common technique for bony procedure is triple arthrodesis. Surgical talectomy can be done in patient with severe equinovarus deformity, however there is few study about this procedure. This study is reported an 18-year-old patient with neglected clubfoot treated with total talectomy.

## 2 Presentation of case

**Fig. 1:**
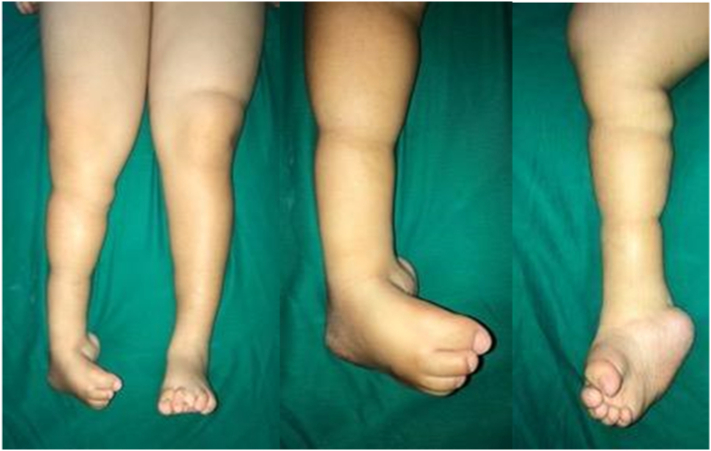
The clinical appearance of the right foot.

**Fig. 2:**
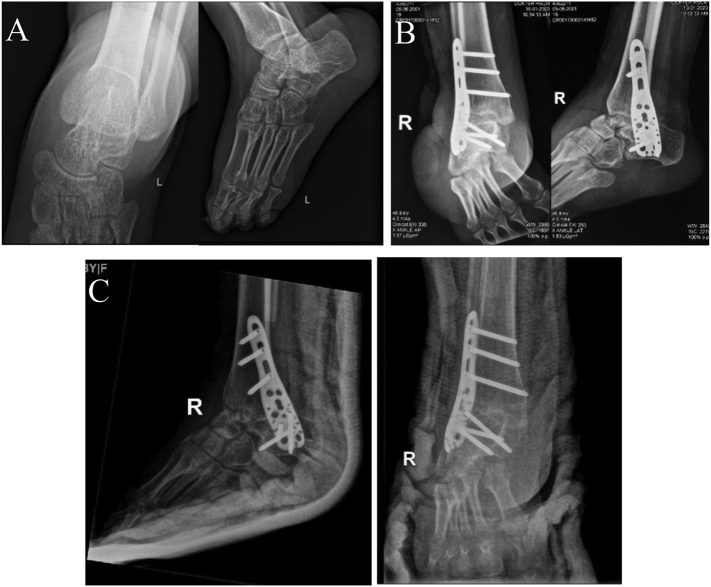
(A) Preop X-ray of the right ankle, (B) post 1st stage deformity correction using Z-plasty, total talectomy, and tibiocalcaneal arthrodesis, (C) post 2nd stage deformity correction using posteriomedial release, tendon lengthening (Tibialis posterior, FDL, FDB) and plantar fascia release.

An 18-year-old female presented with crooked right feet since birth. The deformity was present on her right foot since birth. It caused pain, especially during walking and standing for a long time. Occasionally the deformity resulted in skin infection on the bottom of the crooked foot. At the moment, she could walk in limping gait without walking aid. The patient was born aterm 39 weeks through caesarean delivery due to severe preeclampsia. There was delayed development of walking at 2 years and 9 months. Previously, she had history of spina bifida and undergone surgery by a neurosurgeon in 2001. Afterward, she underwent VP shunt surgery. Physical examination demonstrated cavus varus, tenderness of the right foot, and limited ankle motion. The physical examination revealed equinovarus deformity, with the difficulty in plantarflexion and dorsoflexion of the foot (Fig. 1). Talocalcaneal angle pre-operative was 25^0^ (Fig. 2).

The patient was diagnosed with neglected right neurogenic clubfoot, and she underwent a 2-stage deformity correction consisting of Achilles tendon lengthening using Z-plasty, total talectomy, and tibiocalcaneal arthrodesis followed by posteriomedial release, tendon lengthening (tibialis posterior, Flexor Digitorum Longus (FDL), Flexor Digitorum Brevis (FDB)) and plantar fascia release.

For the first stage of deformity correction, the patient was in left lateral decubitus position and under general anesthesia. After antiseptic and aseptic procedure was performed, posterior incision of tibial area was performed just above the achilles tendon. Lengthening of the tendon was performed using the-Z design for about 7 cm. Posterior lateral malleolus was done and continued by fibular osteotomy around 7 cm from the sindesmosis. Soft tissue was released, and the talus was exposed. We then performed total talectomy and denudation of calcaneal, tibia, and cuboid bone. Philos plate was considered for tibiocalcaneal arthrodesis assisted by C-arm. We applied bone graft from the remnant of the talus. The achilles tendon was sutured and the operation wound was closed layer by layer and then back slab was applied at the level of mid cruris with ankle in plantigrade position.

**Fig. 3:**
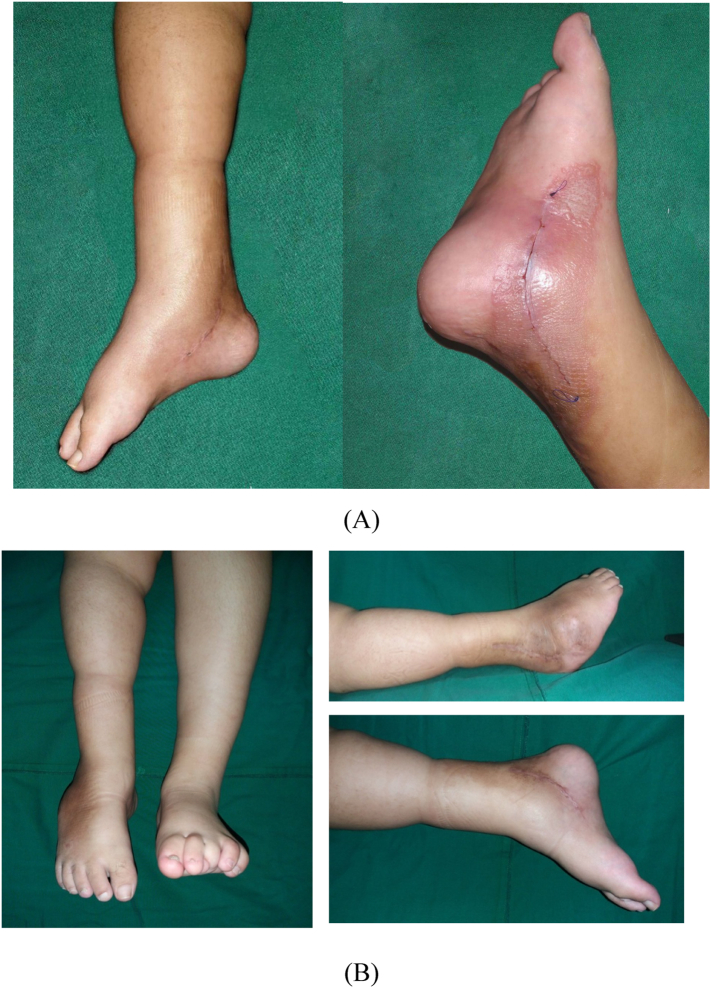
(A) Post operation local state of the right foot (B) post operation follow up.

The second stage of the surgery was performed about 2 months apart. Pre-operation antibiotics of cefoperazone sulbactam was given an hour prior to the operation. The patient was positioned supine and under general anesthesia. Aseptic and antiseptic procedure was performed around the operation site. Curve incision on the posteromedial side of the malleolus was performed. The incision was done until the tendon was exposed. After opening the operation site, we found fibrosis lesion on Flexor Hallucis Longus (FHL), FDL, FDB tendon and the plantar fascia. Z-plasty procedure was performed on FHL, FDL, and FDB and proceed to suture using prolene 4.0. The FDL and the FDB was sutured together to form a “Y” pattern. The plantar fascia was then released. After that, the wound was washed with sterile NaCl 0.9% and the wound was sutured back layer by layer, and back slab was applied (Fig. 3). Talocalcaneal angle post-operative was 30^0^ (Fig. 2).

**Fig. 4:**
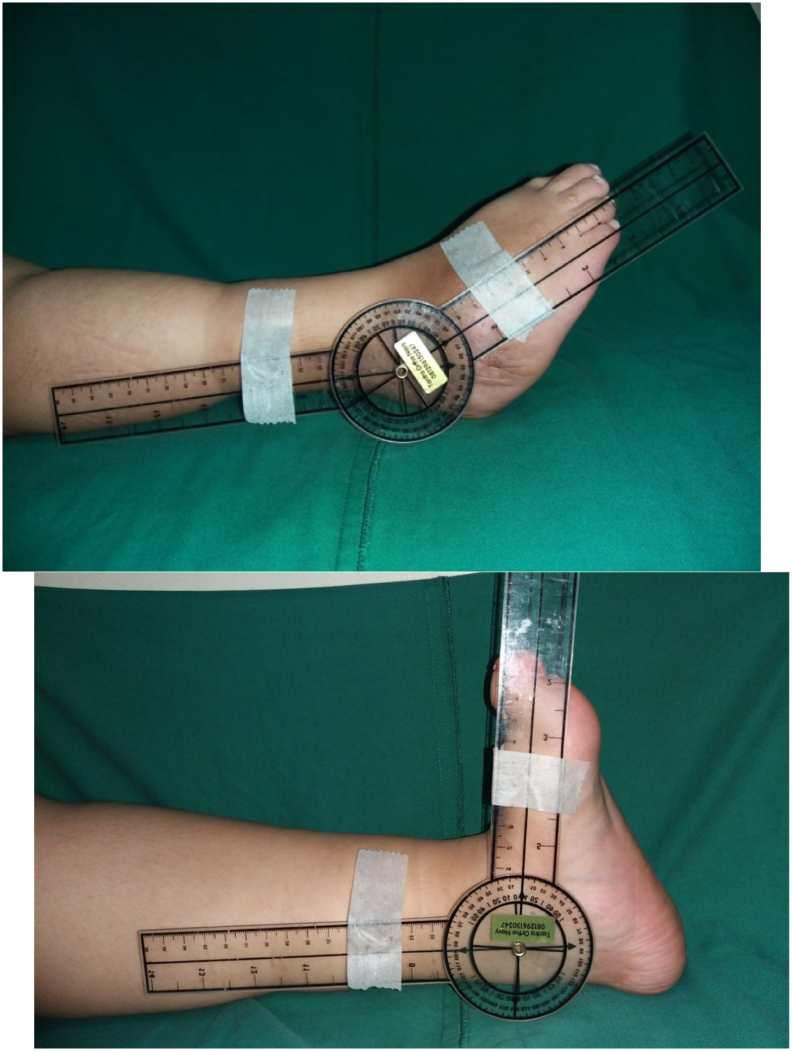
Post operation follow up ROM of Ankle Joint.

**Fig. 5:**
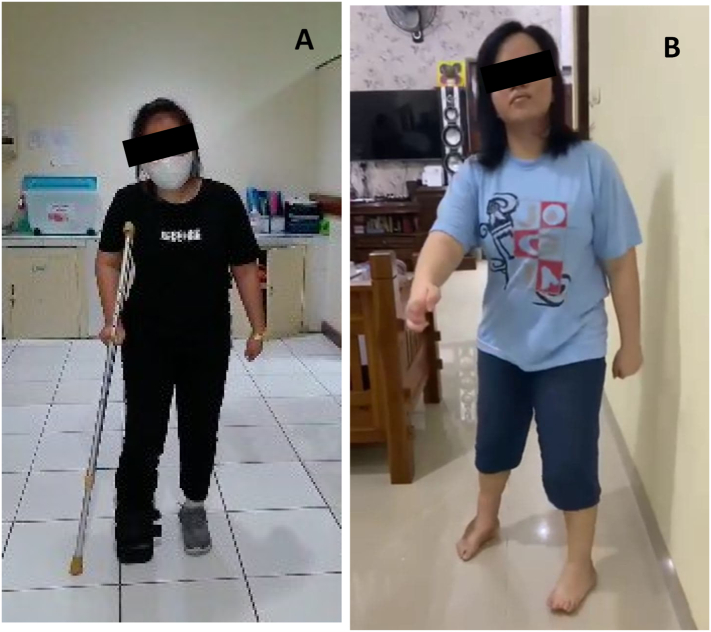
(A) Walking aid, (B) post operative 11 months.

The patient was followed up after the operation. No infection on the wound or dehiscence (Fig. 4). Pain was reduced and no other complain was recorded. On 4 months follow up, the patient had felt that the movement of the ankle was better. The range of motion of the ankle improved and the patient was able to walk using crutch on ipsilateral side with boot orthosis (Fig. 5). This work has been reported in line with SCARE criteria [6].

## 3 Discussion

Clubfoot may appear in isolation, syndromic, or associated with other congenital malformations or deformations. If the clubfoot is left untreated, it may cause severe deformity of the lower limb, with related pain and gait disturbance, often resulting in diminished quality of life and ability to work [7].

Neglected clubfoot can be defined as cases untreated until after a child starts to walk, some studies also suggested to include not having treatment before age 2 or 3. It is often seen commonly in low-income countries where health inequalities and the distances of some rural communities from suitable clinics are large. The neglected clubfoot worsens over time due to the forces transmitted through the malpositioned foot during ambulation. In extreme cases, the foot can face backward, with the patient walking on the dorsum of the foot. Painful callosities develop on the unsuitable dorsal skin of the foot, which then breaks down and becomes infected. Prolonging the neglected clubfoot will make the foot progressively becomes more difficult to shod and to treat [8]. In our case, the patient had deformity on her right foot since birth and came to the clinic at the age of 18. Our patient came without any previous treatment of the foot and the patient also had history of spina bifida. We presumed the clubfoot was associated with congenital deformation. As in literature, the deformity cause pain on the foot especially for walking and standing for a long time. Occasionally it caused skin infection just on the bottom of the crooked foot.

The Ponseti technique for the treatment of congenital clubfoot has been well documented. Although it is best to perform in younger patient. For neglected clubfoot, modifications in the Ponseti method have included greater numbers of serial treatments, longer periods of casting, longer manipulations, and the use of ankle-foot orthoses (AFOs) rather than boots and bars. Encouraging results have been reported for patients up to 12 years of age [9,10]. In our neglected clubfoot case, we did not use any ponseti or modified ponseti technique. There is no literature stating ponseti efficacy in adult neglected clubfoot. We use back slab for immobilization after the first stage surgery.

In untreated clubfoot of a skeletally mature individual, the problem is in achieving correction of a complex combination of deformities, including equinus, varus, and cavus. This almost invariably requires combinations of bony and soft tissue deformities, as well as considerations of tendon transfer, to address the frequently occurring neurologic component of the deformity [11]. In our case, we performed both of the bone and soft tissue procedure for this patient, which was Z-plasty, total talectomy, and tibiocalcaneal arthrodesis followed by posteriomedial release, tendon lengthening and plantar fascia release. Talectomy itself has been described as a way of managing clubfoot in spina bifida and arthrogryposis [12,13]. It has also used as the treatment for neurogenic clubfoot. Talectomy provide the laxity to correct the deformity, but often leads to a significant limb length deformity and distorted anatomy [3]. In our case, post-operative examination revealed that there was no limb length discrepancy. This procedure gave excellent outcome to the gait evaluation analysis [5].

There are other treatment options for the older child or adolescent with a rigid clubfoot. If an extended treatment course is impossible, triple arthrodesis is the most expeditious way to gain correction with the fewest number of complications. The foot growth is not affected any more compared to osteotomy procedures [4]. Modified lambrinudi triple arthrodesis is more preferred. The Lambrinudi triple arthrodesis has been reported a successful use in a patient with clubfoot deformity of neurogenic origin [3]. The Ilizarov external fixator has become a good alternative in treating neglected clubfoot. Recent literature shows good results using the distraction-osteogenesis of Ilizarov for the treatment of such rigid and chronic deformities. The Ilizarov method is believed to be safer than other more extensive operative procedures of neglected clubfoot. It allows simultaneous correction of all foot deformities associated with minimal surgery, reducing risks of cutaneous or neurovascular complications and avoiding excessive shortening of the foot [9]. It also can be used for residual idiopathic or neurogenic clubfeet cases [14].

Due to the dynamic 4-dimensional nature of the condition, neglected, chronic, or relapsed clubfoot is a major treatment challenge. Complete restoration of normal anatomy is prevented by the congenital origin of the condition; the emphasis is therefore on achieving pain-free operation rather than radiographic or cosmetic perfection. Incremental changes in foot form and joint alignment, such as the Ponseti and Ilizarov, are favoured by less invasive approaches. Open surgery with or without osteotomy can then be limited to certain moderately stiff club feet in patients who are not appropriate for Ilizarov care or who are unlikely to be completely corrected by the Ponseti technique [8]. Up to our knowledge, no similar case report using this technique was published before, hence we couldn't compare our outcome with other published studies.

During the follow up, examinations showed excellent results with no sign of infection on the wound or dehiscence. Range of motion of the ankle joint improved and there was no complained of post-operative pain. At first week patient can walk independently using a crutch with no complain of pain, after few weeks she can walk independently with no crutch by using foot-ankle orthoses without difficulty. Overall, the patient was satisfied with the post-operative results.

## 4 Conclusion

Two stage deformity correction consisting of achilles tendon lengthening using Z-plasty, total talectomy, and tibiocalcaneal arthrodesis followed by posteriomedial release, tendon lengthening (Tibialis posterior, FDL, FDB) and plantar fascia release, can be successful in patients with neglected neurogenic clubfoot. Further studies are required to investigate the safety and efficacy of such procedures in neurogenic clubfoot compared with other procedure.

## Consent for publication

Written informed consent was obtained from the patient for publication of this case report and accompanying images. A copy of the written consent is available for review by the Editor-in-Chief of this journal on request.

## Ethical approval

Ethical approval was not required in the treatment of the patient in this report.

## Funding

This research did not receive any specific grant from funding agencies in the public, commercial, or not-for-profit sectors.

## Author contribution

Ihsan Oesman contributes in the study concept or design, data collection, analysis and interpretation, oversight and leadership responsibility for the research activity planning and execution, including mentorship external to the core team.

Cynthia Mutiara contributes to the study concept or design, data collection and writing the paper.

## Guarantor

Ihsan Oesman, MD.

## Research registration number

Does not need any registration.

## Provenance and peer review

Not commissioned, externally peer-reviewed.

## Disclaimer

No patient or author details are included in the figures.

## Declaration of competing interest

The authors declare no conflicts of interest.
